# Introducing Mg-4Zn-3Gd-1Ca/ZnO nanocomposite with compressive strengths matching/exceeding that of mild steel

**DOI:** 10.1038/srep32395

**Published:** 2016-08-30

**Authors:** Y. Chen, S. Tekumalla, Y. B. Guo, M. Gupta

**Affiliations:** 1 Department of Mechanical Engineering, National University of Singapore, 9 Engineering Drive 1, Singapore 117576, Singapore.

## Abstract

This work introduces Mg-4Zn-3Gd-1Ca/2ZnO (wt.%) nanocomposite fabricated using the technique of disintegrated melt deposition and extrusion. Addition of ZnO nanoparticles enhanced the compressive strengths of alloy by ~100 MPa. Nanocomposite samples display high strength and good ductility: 0.2% compressive yield stress of 355 MPa, ultimate compressive stress of 703 MPa, and compressive failure strain of 10.6%. The significant enhancement of compressive yield stress is mainly attributed to the grain refinement by adding nanoparticles. The strength levels exceed that of commercial magnesium alloys (i.e. WE43, WE54, ZK60, and ME21) and mild steels (i.e. S275 and S355), making Mg-4Zn-3Gd-1Ca/2ZnO a very promising material for multiple engineering and biomedical applications.

Magnesium is the lightest structural metal – only two thirds the density of aluminum. The light weight of Mg and its alloys endows them with tremendous potential for weight reduction in transportation sector including reduction in green house emission and energy consumption. However, the low strength and poor ductility at room temperature of Mg alloys hinder their engineering applications. Adding rare earth (RE) elements into Mg is one effective way to improve mechanical properties^1^. The extruded alloy Mg-1Zn-2.5Gd (at.%) reported by Yamasaki *et al.*^2^ exhibited a good balance of tensile yield strength (345 MPa) and elongation (6.9%) due to refined grains and well dispersed long period stacking ordered (LPSO) phase. Rolled Mg-1Zn-Gd (2 and 3 wt.% for Gd) sheet studied by Wu *et al.*^3^ displayed a high tensile failure strain (~50%) as a result of texture weakening by adding Gd. Addition of Ca element into Mg alloys can also improve strength effectively through grain refinement. Extruded Mg-5.25Zn-0.6Ca (wt.%) alloys presented by Tong *et al.*^4^ and Mg-1.8Zn-0.3Ca (at.%) presented by Somekawa and Mukai^5^ showed good tensile yield strength (~300MPa) with ultrafine grains. Recently, work done by Wen *et al.*^6^ showed that with increasing the amount of Ca element (0–1 wt.%), the ultimate tensile strength of extruded Mg-4Zn-2Gd-Ca alloys increased. With increase of Gd addition (0–2.52 wt.%), the creep properties of as-cast Mg-3.8Zn-2.2Ca-Gd alloys were improved^7^. However, reports on alloy Mg-Zn-Gd-Ca and compressive properties are extremely limited^4^.

Addition of nano-size reinforcements to Mg alloys is another effective way to improve the mechanical properties beyond that of alloying elements. Studies by Nguyen *et al.*^8^ and Chen *et al.*^9^ demonstrated that addition of Al_2_O_3_ nanoparticles into AZ31 improved tensile ductility and strength simultaneously, while the compressive strength was also enhanced. Similar results were reported for adding ZnO nanoparticles to Mg matrix^10^.

In literature, some studies^11^,^12^ estimated that the transition from twinning to slip may happen when the grain size of magnesium alloy is smaller than 3 μm. However, the results from work by Li *et al.*^13^ for AZ60 with grain size of 0.8 μm and work by Chen *et al.*^14^ for AZ31-based nanocomposite with grain size of 1.5 μm, showed that tension twinning remained the predominant deformation mode for the initial plastic flow under uniaxial compression. Results that are available in open literature remain contradictory. Thus, it needs further research work. Accordingly, the present study investigates the effect of addition of ZnO nanoparticles in a new Mg-Zn-Gd-Ca alloy emphasizing particularly on the compressive response.

## Results

### Compressive mechanical properties and microstructure

**Figure 1:**
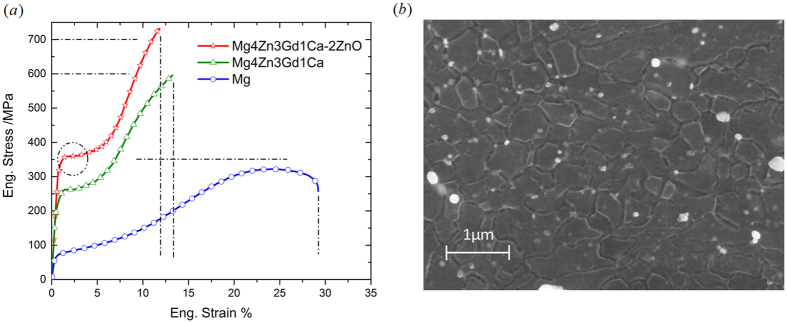
Mechanical properties and grain morphology. (**a**) Engineering stress-strain curves for compression on three materials; (**b**) FESEM image for grain morphology of the nanocomposite.

**Figure 2:**
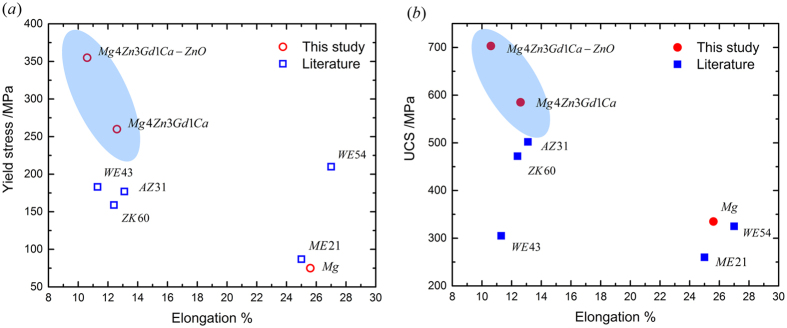
Comparison of compressive properties of the materials in this study and in literature. (**a**) Compressive yield stress against elongation; (**b**) ultimate compressive stress against elongation.

Figure 1(a) shows typical engineering stress-strain plots of three materials subjected to quasi-static compression. Compressive mechanical properties and grain size of three materials studied are given in Table 1. Compared to pure Mg, the alloy Mg4Zn3Gd1Ca exhibits high strength and good ductility: 0.2% compressive yield stress *σ*_*YS*_ of 260 MPa, ultimate compressive stress *σ*_*UCS*_ of 585 MPa and failure strain *ε*_*FS*_ of 12.6%. The 0.2% compressive yield stress of the alloy is almost 3.5 times that of pure Mg. Furthermore, addition of 2 wt.% ZnO nanoparticle into the alloy enhanced the strength by ~100 MPa; *σ*_*YS*_ of 355 MPa, *σ*_*UCS*_ of 703 MPa were obtained. In addition, failure strain of the nanocomposite is 10.6%, only slightly lesser than that of the alloy. It indicates that addition of nanoparticles can maintain the ductility of the alloy, while it increases strength significantly. Figure 2 presents the comparison of compressive mechanical properties of the materials in this study and the extruded Mg alloys in literature; Table 1 gives the corresponding specific values. The compressive yield stress of mild steel S275 and S355 is also given in Table 1. It is evident that with a comparable elongation, the nanocomposite Mg4Zn3Gd1Ca-2ZnO exhibits significant higher strength than other extruded Mg alloys shown in Fig. 2, and the compressive yield strength levels either matches or surpasses that of mild steel.

Compared to pure Mg, significant grain refinement was observed in the alloy Mg4Zn3Gd1Ca. The grain size reduces from 24. 6 μm for Mg to 1.42 μm for the alloy. Further reduction of grain size was achieved by addition of ZnO nanoparticle – grain size of the nanocomposite is ~0.9 μm, ~60% that of the alloy, as shown in Fig. 1(b).

### XRD results

**Figure 3:**
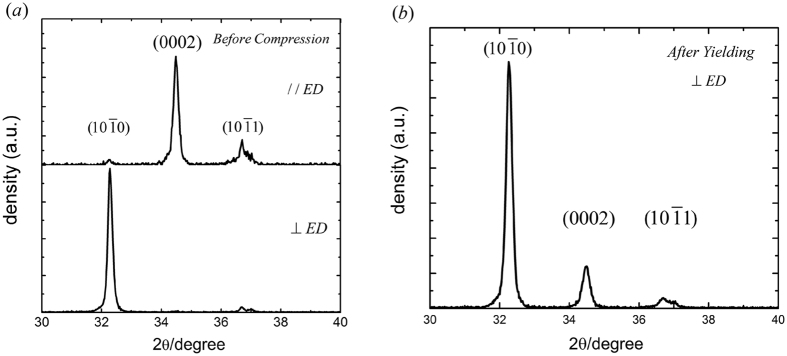
X-ray diffraction patterns of the nanocomposite. (**a**) Before compression; (**b**) after yielding (at a strain of 2%). (ED: extrusion direction).

Two types of specimens of three materials – before compression and after yielding (at a strain of 2%) – were subjected to XRD scanning. The results for the nanocomposite are given in Fig. 3. In Fig. 3(a), corresponding to the basal plane (0002), the highest density peak is observed for scanning along the extrusion direction and the negligible density peak for scanning perpendicular to the extrusion direction. This indicates that specimens of the nanocomposite have a typical basal texture, as commonly observed for the extruded magnesium-based material. In Fig. 3(b), after yielding (at a strain of 2%), significant increment of peak density for the basal plane (0002) is observed for scanning perpendicular to the extrusion direction. This is attributed to activation of 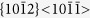 tension twinning, because this twinning mode can rotate the grain lattice by ~90^o^. The fraction of basal plane perpendicular to the extrusion direction can be expected to increase with activation of this twinning mode. In addition, the low strain hardening for the initial plastic flow, as highlighted by the circle in Fig. 1, also indicates the activation of 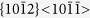 tension twinning. Therefore, although the nanocomposite has ultrafine grains (~0.9 μm), tension twinning still plays a dominant role in the initial stage of plastic flow. Similar XRD results were obtained for the alloy and pure Mg. For brevity, these XRD spectrums are not presented.

## Discussion

**Figure 4:**
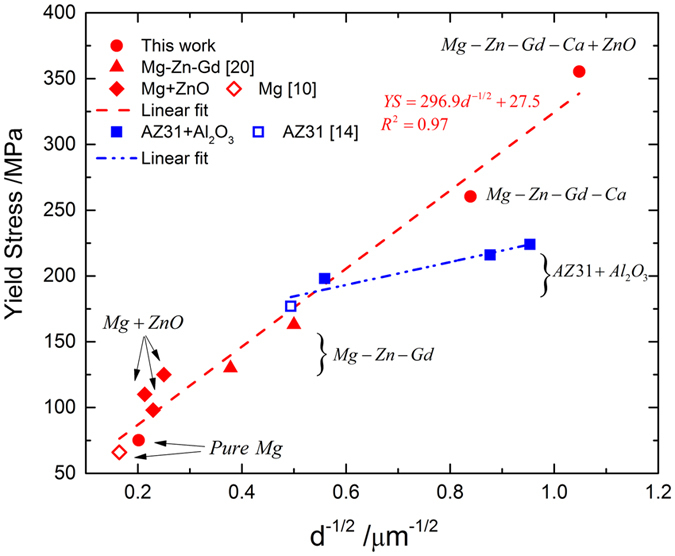
Hall-Petch Relation. Plots of compressive yield stress against inverse of square root of grain size for Mg-based materials synthesized by DMD method.

The well-known Hall-Petch relation^19^ defines the relationship between grain size and yield stress for polycrystalline materials as follows:


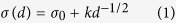


where *σ* is the 0.2% yield stress; *σ*_0_ is a material constant, *k* the strengthening coefficient and *d* is the grain size. Figure 4 shows the relationship of *σ* and *d*^−1/2^ for Mg-based materials. All materials in the figure were fabricated by DMD method and extruded with the same extrusion ratio (20.25:1).

The red marks, from the left bottom corner to the right up corner, denote respectively: pure Mg, Mg reinforced with ZnO nanoparticles^10^, Mg added with alloying elements – Zn and Gd^20^, Mg added with alloying elements – Zn, Gd and Ca, and Mg containing both alloying elements – Zn, Gd and Ca, and ZnO nanoparticles. All the data fitted very well into a linear line because the value of fitting parameter R-square is 0.97. In Fig. 4, several salient points can be noted: (1) significant enhancement (~100 MPa) of yield stress by addition of ZnO nanoparticles is mainly due to grain refinement; (2) as discussed for Fig. 3, when the grain size reduces to 0.9 μm, tension twinning still plays a predominant role for yield stress. That is to say, the activation stress of tension twinning follows Hall-Petch relation even when the grain is significantly refined; (3) the blue squares in Fig. 4 denote results for Mg Alloy AZ31 and its nanocomposites^14^, which is reinforced by Al_2_O_3_ nanoparticles. It is apparent that the yield stress of these materials follows Hall-Petch relation, as indicated by the blue linear fitting line. However, the slope (or the strengthening coefficient k) of the blue line is much smaller than that of the red line. It means that the combination of alloy elements – Zn and Gd, generates much higher slope than that of Zn and Al. This notable contrast demonstrates that alloying elements display varied strengthening effect on the activation stress of tension twinning. The fundamental mechanism for the discrepancy needs further investigation.

In summary, an alloy Mg4Zn3Gd1Ca and its nanocomposite Mg4Zn3Gd1Ca-2ZnO were synthesized by DMD method followed by hot extrusion. Addition of 2 wt.% ZnO nanoparticles into the alloy generates significant strength enhancement – i.e. ~100 MPa, and maintains good ductility; *σ*_*YS*_ of 355 MPa, *σ*_*UCS*_ of 703 MPa and *ε*_*FS*_ of 10.6% were obtained. Significant increment in strength by adding ZnO nanoparticles is mainly attributed to its grain refinement effect. The XRD results and compressive stress-strain curves demonstrate that at initial stage of plastic flow, tension twinning plays a predominant role for the ultrafine-grained nanocomposite. Superior-performance nanocomposite presented in this study has the capability to replace commercial magnesium alloys and certain mild steels when compressive strength is the material selection criterion.

## Methods

### Materials synthesis

An alloy Mg-4Zn-3Gd-1Ca (wt.%) and its nanocomposite Mg-4Zn-3Gd-1Ca-2ZnO (ZnO nanoparticle with diameters in 90–200 nm) were synthesized by a disintegrated melt deposition (DMD) technique^9^, which includes melting, mixing, disintegrating and deposition steps. Pure Mg, as a reference, was synthesized using the same technique. The cast ingots with 36 mm in diameter and 45 mm in length were homogenized at 400 °C for 4 hours and extruded at 300 °C into 8 mm diameter rods.

### Mechanical testing and microstructure characterization

The 6 mm diameter cylinder specimens with a length-to-diameter ratio of 1, were prepared for room temperature uniaxial compression tests at a strain rate of 5 × 10^−3 ^s^−1^, using an Instron 8874 universal testing machine. An extensometer was used to measure plastic deformation. Three specimens were tested for each material. The microstructures of three materials were observed using an Olympus optical microscope and a field emission scanning electron microscope (FESEM). X-ray diffraction (XRD) analysis was carried out on specimens before compression and after yielding, using an automated Shimadzu Lab-X XRD-6000 diffractometer (Cu Kα, λ = 1.54056 Ǻ) operating at a scanning speed of 2 deg/min. The specimens were etched with a mixture of 1 g oxalic, 1 ml nitric acid, 1 ml acetic acid and 150 ml distilled water.

## Additional Information

**How to cite this article**: Chen, Y. *et al.* Introducing Mg-4Zn-3Gd-1Ca/ZnO nanocomposite with compressive strengths matching/exceeding that of mild steel. *Sci. Rep.* **6**, 32395; doi: 10.1038/srep32395 (2016).

## Figures and Tables

**Table 1:** Compressive mechanical properties and grain size of three materials in this study and other materials in literature.

Materials	Compressive yieldstress (MPa)	UCS (MPa)	FS (%)	Grain size (μm)
Mg	75.2 ± 3.6	335.4 ± 13.8	25.6 ± 2.2	24.6 ± 4.0
Mg4Zn3Gd1Ca	260.5 ± 2.9	585.4 ± 18.9	12.6 ± 0.3	1.42 ± 0.20
Mg4Zn3Gd1Ca-ZnO	355.4 ± 5.0	703.4 ± 39.8	10.6 ± 0.4	0.91 ± 0.47
ME21^15^ (extruded)	87	260	25	NR
ZK60^16^ (extruded)	159	472	12.4	NR
WE43^17^ (extruded)	183	305	11.3	NR
WE54^15^ (extruded)	210	325	27	NR
S275^18^ (≤16 mm plate)	275	NR	NR	NR
S355^18^ (≤16 mm plate)	355	NR	NR	NR

^*^NR denotes not reported.
